# Evaluation and management of obstetric genital fistulas treated at a pelvic floor centre in Germany

**DOI:** 10.1186/s12905-021-01175-x

**Published:** 2021-02-05

**Authors:** Christl Reisenauer, Bastian Amend, Claudius Falch, Harald Abele, Sara Yvonne Brucker, Jürgen Andress

**Affiliations:** 1 Department of Obstetrics and Gynaecology, University Hospital Tübingen, Calwerstrasse 7, 72076 Tübingen, Germany; 2 Department of Urology, University Hospital Tübingen, Hoppe-Seyler-Str. 3, 72076 Tübingen, Germany; 3 Department of General, Visceral and Transplant Surgery, University Hospital Tübingen, Hoppe-Seyler-Str. 3, 72076 Tübingen, Germany

**Keywords:** Faecal incontinence, Obstetric genital fistula, Rectovaginal fistula, Urethro-vaginal fistula, Urinary incontinence, Utero-vaginal fistula, Vesico-vaginal fistula

## Abstract

**Background:**

Obstetric genital fistulas are an uncommon condition in developed countries. We evaluated their causes and management in women treated at a German pelvic floor centre.

**Methods:**

Women who had undergone surgery for obstetric genital fistulas between January 2006 and June 2020 were identified, and their records were reviewed retrospectively.

**Results:**

Eleven out of 40 women presented with genitourinary fistulas, and 29 suffered from rectovaginal fistulas. In our cohort, genitourinary fistulas were more common in multiparous women (9/11), and rectovaginal fistulas were more common in primiparous women (24/29). The majority of the genitourinary fistulas were at a high anterior position in the vagina, and all rectovaginal fistulas were at a low posterior position. While all genitourinary fistulas were successfully closed, rectovaginal fistula closure was achieved in 88.65% of cases. Women who suffered from rectovaginal fistulas and were at high risk of recurrence or postoperative functional discomfort and desired another child, we recommended fistula repair in the context of a subsequent delivery. For the first time, pregnancy-related changes in the vaginal wall were used to optimize the success rate of fistula closure.

**Conclusions:**

In developed countries, birth itself can lead to injury-related genital fistulas. As fistula repair lacks evidence-based guidance, management must be tailored to the underlying pathology and the surgeon’s experience. Attention should be directed towards preventive obstetric practice and adequate perinatal and postpartum care. Although vesicovaginal fistulas occur rarely, in case of urinary incontinence after delivery, attention should be paid to the patient, and a vesicovaginal fistula should be ruled out.

*Trial registration *Retrospectively registered, DRKS 00022543, 28.07.2020.

## Background

As a result of nationwide access to modern medicine, obstetric genital fistulas (OGF) are an uncommon condition in developed countries. Due to the unrestricted availability of caesarean sections, obstructed labour no longer leads to genital fistulas in Germany. Nevertheless, birth itself can result in injury-related genital fistulas.

As genital fistula repair lacks evidence-based guidance, management must be tailored to the underlying pathology and the surgeon’s experience [1]. The aim of this retrospective study was to evaluate the causes and management of OGF in women treated at a pelvic floor centre in a developed country between January 2006 and June 2020. Furthermore, we aimed to share our practices and experience with other surgeons who care for women with urinary or faecal incontinence due to obstetric fistulas.

## Methods

Women undergoing surgery for OGF between January 2006 and June 2020 at the Department of Obstetrics and Gynaecology in Tübingen, Germany, were identified, and their records were reviewed retrospectively. According to the ICD-10 codes (N82.0, N82.1, N82.3) the OGF were extracted from the digital patients file (SAP® clinical documentation system). The collected data included patient age and obstetric history, fistula aetiology, location, size, management and outcomes.

In our present publication, we included all 40 OGF patients treated at our department between January 2006 and June 2020. We included 4 vesicovaginal fistulas (VVFs) and 12 rectovaginal fistulas (RVFs) [2, 3] from two previous publications and presented 24 new cases. The previous studies were designed with separate goals in mind. In the present publication, we describe all genitourinary fistulas and present a new approach for the management of obstetric rectovaginal fistulas. Furthermore, we compare obstetric fistulas in a developed country to those in developing countries. As obstetric fistulas still occur in developed countries and almost every birth-related fistula has different characteristics, we consider the presentation of a high number of different obstetric fistulas very important.

OGF was diagnosed from history and by physical examination, urethrocystoscopy, hysteroscopy and rectoscopy. As obstetric fistulas are a heterogeneous group and their repair lacks evidence-based guidance, we tailored the repair to the specific anatomical defect. If the fistula was tethered so high that its upper edge could not be reached transvaginally, repair took place via the abdominal route or a combined approach. Regarding timing, the fistula repair was performed after the resolution of the local inflammation, infection and oedema of the tissue surrounding the fistula, approximately 3 months after diagnosis.

The surgical technique used for genitourinary fistula (GUF) closure was fistula excision and tension-free multilayer closure. Martius flaps, omentum majus flaps and bioimplant interposition were used for large, recurrent or residual GUFs. Urethra reconstruction was performed with a graft from the labium minus (Table 2). All patients received perioperative antibiotics and a suprapubic catheter for three weeks. Ureteral stents were placed intraoperatively for 5 weeks in cases in which the fistulas were located close to the ureteric orifices.

For RVF closure, the following surgical techniques were used: fistulectomy and tension-free multilayer closure, fistulectomy and tension-free multilayer closure with Martius flap interposition, conversion to a fourth-degree perineal tear, ligation of intersphincteric fistula tract (LIFT) procedure and transanal rectal-mucosa flap.

A temporary protective stoma for the diversion of the faecal stream was created in women with a large, recurrent or persistent RVF.

In women with RVF with a very thin perineum, very poor tissue condition, and a narrow vagina who were consequently at a high risk of recurrence or postoperative functional discomfort (e.g., vaginal stenosis, dyspareunia) and desired another child, we recommended and performed fistula repair in the context of a subsequent delivery.

The patients underwent full bowel preparation preoperatively, with the exception of the pregnant women, who received two enemas. Postoperative management comprised dietary measures for 5 days and antibiotics for 3–5 days. Avoidance of constipation was also important. Retrocession of the ostomy was carried out approximately three months postoperatively after healing had been confirmed. All patients were advised to abstain from sexual intercourse for three months.

### Statistical analyses

The data are presented descriptively and considered in the context of the current literature.

## Results

In total, 40 women with OGF were referred to the Department of Obstetrics and Gynaecology Tübingen between January 2006 and June 2020. Eleven (27.5%) of the 40 women presented with GUF, and 29 (72.5%) out of 40 suffered from RVF. Three women with RVF delivered at our hospital.

### Presentation and management of obstetric GUF

The GUF group comprised patients with VVF (2/11), vesico-vaginal fistulas with involvement of the cervix uteri (3/11), vesico-uterine fistulas (4/11), a vesico-utero-vaginal fistula (1/11) and a urethro-vaginal fistula (1/11). The patients’ characteristics are summarized in Tables 1 and 2.

**Table 1:** Patient characteristics

Type of obstetric fistula	Number of fistulas	Age at time of fistula diagnosis (years)	Parity				Fistula size (mm)	Mode of delivery leading to fistula formation		
GUF	11	26—66 (average 36.18)	Primiparaous		Multiparaous		3 – 40	Spontaneous delivery	Forceps assisted delivery	Cesarean section
			2		9			3	1	7
RVF	29	21 – 38 (average 29.27)	Primi- paraous	First vaginal delivery	Multi- paraous	One to three previous vaginal deliveries	2 – 40	Spontaneous delivery	Forceps assisted delivery	Vacuum assisted delivery
			24	26	5	3		20	2	7

*GUF* genitourinary fistula, *RVF* rectovaginal fistula

**Table 2:** Characteristics of patients with genitourinary fistulas

Patient number	Type of urogenital fistula and fistula characteristics	Mode of delivery leading to fistula formation	Age at time of fistula diagnosis (years) and parity	Previous treatment	Fistula treatment in our department
1	VVF Diameter: 3 mm Location: vaginal apex/vesical trogone	07/2015 Caesarean section and total hysterectomy due to placenta previa percreta	36, 3 para	09/2015 abdominal fistula excision and closure with peritoneal flap interposition	03/2016 vaginal fistula excision, tension-free multilayer closure
2	VVF Diameter: 20 mm Location upper third of the anterior vaginal wall close to the cervix/vesical trigone	09/2014 caesarean section with intraoperative bladder injury	35, 2 para	09/2014 abdominal VVF closure	01/2015 abdomino-vaginal fistula excision and tension-free multilayer closure with omentum majus flap interposition
3	VVF with involvement of the cervix uteri Diameter: 30 mm Location: upper third of the anterior vaginal wall and cervix/vesical trigone	11/2017 caesarean section with intraoperative bladder injury	29, 2 para		Ureteral stents placement 04/2018 vaginal fistula excision, tension-free multilayer closure and Martius- Flap interposition 06/2018 abdomino-vaginal rest fistula (2 mm) excision and closure using an omentum majus flap
4	VVF with involvement of the cervix uteri Diameter: 40 mm Location: upper third of the anterior vaginal wall and cervix/vesical trigone	03/2016 caesarean section	33, 2 para		Ureteral stents placement 07/2016 abdomino-vaginal fistula excision and closure with concomitant hysterectomy using an omentum majus flap 01/2017 vaginal rest-fistula (5 mm) excision and closure with Martius-Flap interposition
5	VVF with involvement of the cervix uteri Diameter: 20 mm Location: upper third of the anterior vaginal wall and cervix/posterior bladder wall	12/2011 forceps assisted delivery	36, 2 para		01/2012 vaginal tension-free multilayer closure and cervix reconstruction 04/2012 abdomino-vaginal rest-fistula (3 mm) excision and closure with an omentum majus flap
6	Vesico-uterine fistula Diameter: 3 mm Location: supratrigonal/upper third of the right cervical wall	Spontaneous delivery 1982	66, 2 para		06/2020
7	Vesico-uterine fistula Diameter: 15 mm Location: posterior baldder wall/cervix	07/2018 caesarian section and supracervical hysterectomy (due to haemorrhage) and bladder injury	33, 2 para		10/2018 abdomino-vaginal tension free multilayer fistula closure with removal of the cervix and omentum majus flap interposition
8	Vesico-uterine fistula Diameter: 30 mm Location: posterior bladder wall/isthmus uteri	2011 caesarian section	37, 3 para (the fistula was diagnosed in pregnancy)		02/2017 caesarian section and abdominal tension free multilayer fistula closure with bioimplant (Serasis Firma Cook) interposition
9	Vesico-uterine fistula Diameter: 3 mm Location: posterior bladder wall/isthmus uteri	8/2016 caesarian section with bladder injury	38, 1 para		06/2017 vaginal fistula excision and tension free multilayer fistula closure with martius-flap interposition
10	Vesico-utero-vaginal fistula Diameter 30 mm Location: upper third of the anterior vaginal wall/anterior wall of the uterus above the cervix/vesical trigone	Spontaneous delivery	29, 1 para		Ureteral stents placement 11/2019 abdomino-vaginal fistula excision and tension-free multilayer closure of the bladder, uterus and vagina with omentum majus flap interposition
11	Urethro-vaginal fistula Diameter: 25 mm	2016 Spontaneous delivery	26, 2 para		11/2019 Urethra reconstruction (using a graft from labium minus) and Martius-flap interposition

*VVF* vesico-vaginal fistula

Nine out of 11 GUFs were diagnosed after delivery; the exceptions were two vesico-uterine fistulas. One fistula remained unrecognized for 38 years, and the other was diagnosed six years later during a subsequent pregnancy [4]. Two out of seven caesarean sections were performed simultaneously with a total hysterectomy and a supracervical hysterectomy due to postpartum haemorrhage. In four cases, bladder injury occurred during surgery. Nine of the 11 GUFs were primary fistulas, and two were recurrent fistulas that occurred after one previous attempt at repair.

**Fig. 1:**
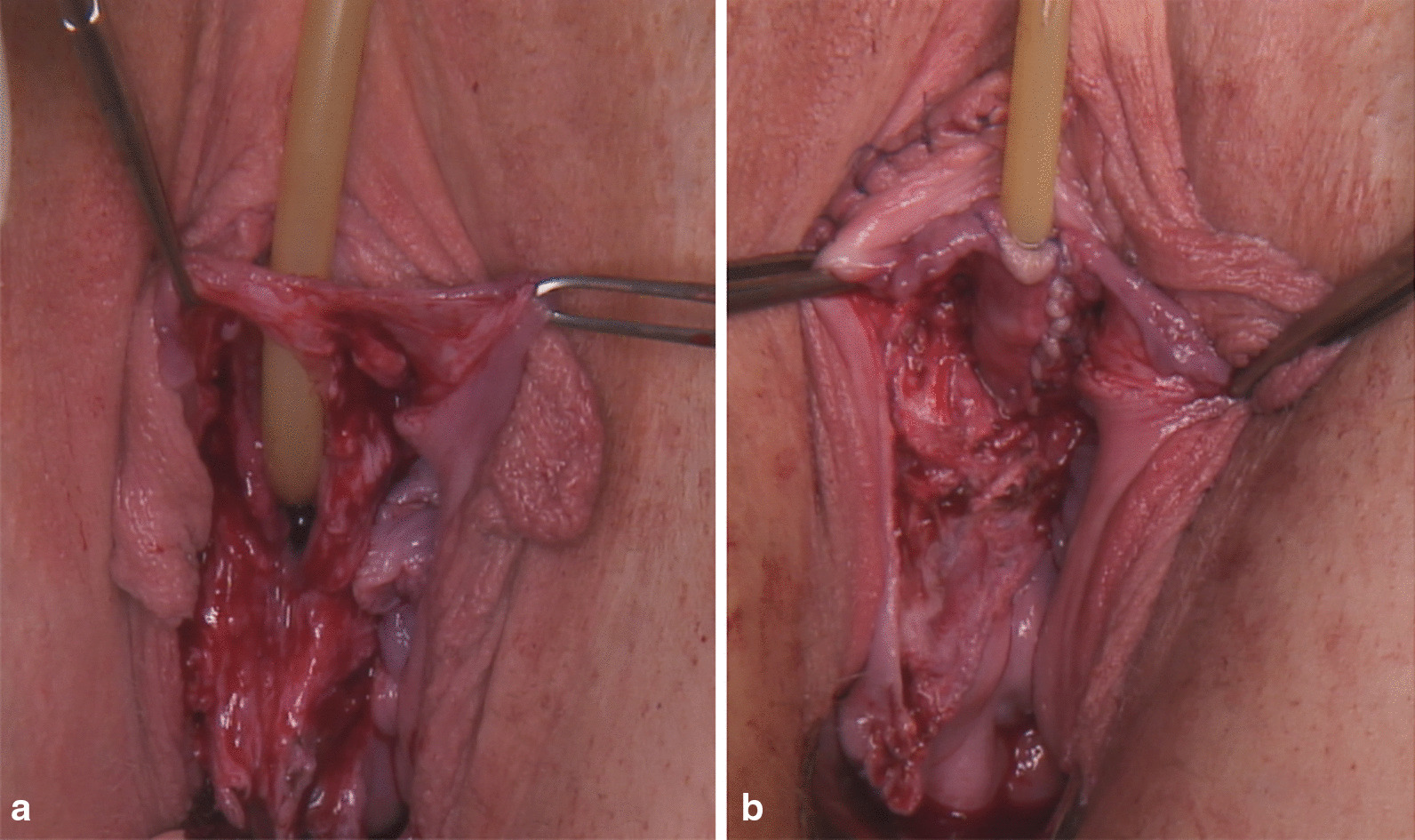
**a** Urethro-vaginal fistula after spontaneous delivery; **b** Urethra reconstruction with a labium minus graft

The urethro-vaginal fistula was closed on the first attempt by reconstruction of the urethra using a graft from the labium minus covered by a Martius flap (Fig. 1a, b).

**Fig. 2:**
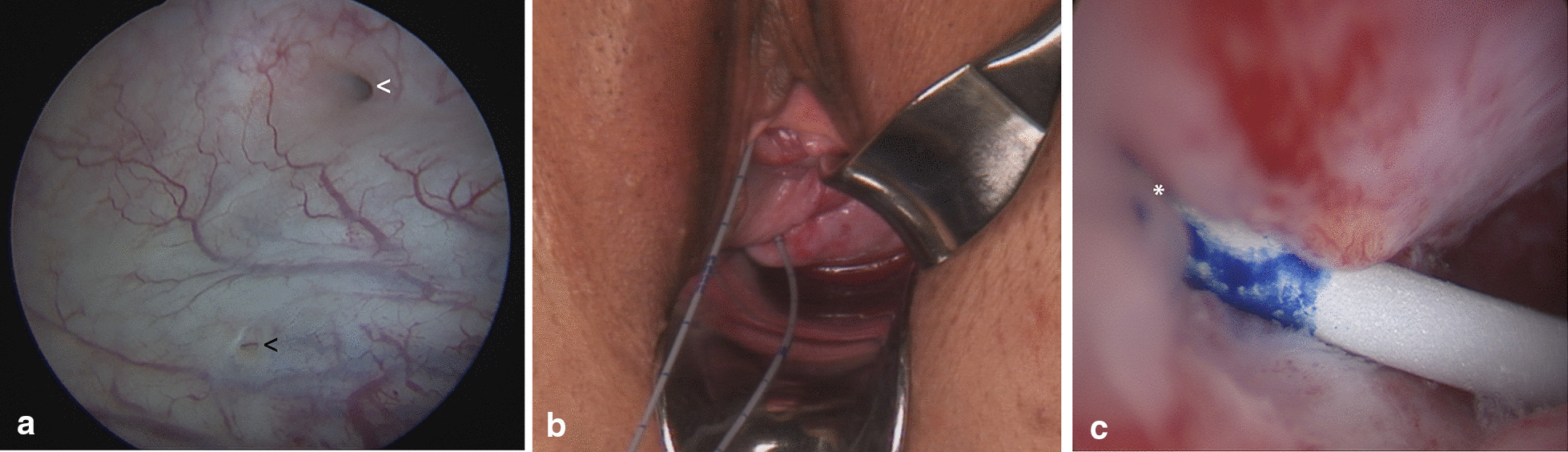
Utero-vesical fistula; **a** cystoscopic view: the white arrow shows the fistula, and the black arrow shows the right ureteric orifice; **b** the inserted catheter runs through the urethra, bladder, utero-vesical fistula, cervix and vagina; **c** hysteroscopic view: the catheter passes through the vesico-uterine fistula into the cervical canal. The fistula is marked with a white asterisk and is located at the upper third of the right cervical wall

Two out of four vesico-uterine fistulas (Fig. 2a–c) were closed vaginally, one was closed abdomino-vaginally, and one was closed abdominally; all were closed on the first repair attempt.

**Fig. 3:**
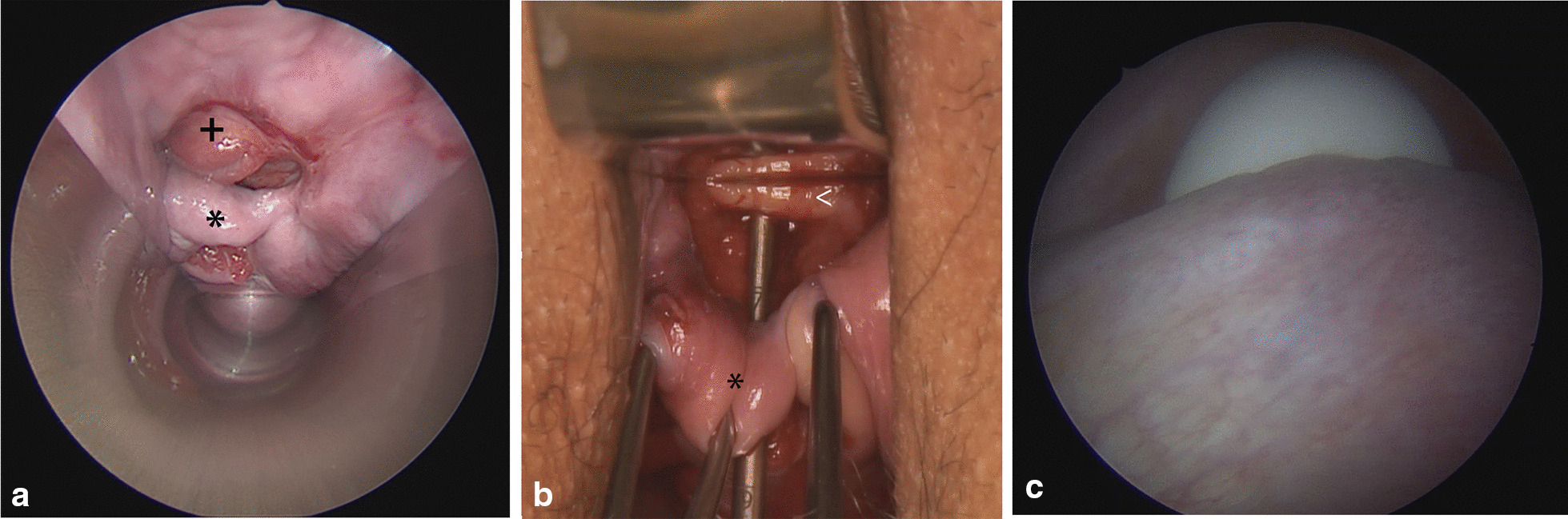
Vesico-utero-vaginal fistula after spontaneous delivery; **a** vaginal view: the black asterisk shows the cervix, and the black plus sign shows the bladder wall everted through the fistula into the vagina; **b** vaginal view after the introduction of a uterine probe: the anterior part of the cervix (black asterisk) is detached from the corpus uteri (white arrow); **c.** cystoscopic view of the fistula covered by the finger during a simultaneous vaginal examination

The vesico-utero-vaginal fistula (Fig. 3a–c) was also repaired on the first attempt.

Three out of five vesico-vaginal fistulas with or without involvement of the cervix were closed after two attempts via abdomino-vaginal and vaginal approaches, and two out of five were closed on the first attempt, vaginally in one case and abdomino-vaginally in the other (Table 2).

The postoperative period was uneventful, and all GUF were closed successfully.

### Presentation and management of obstetric RVF

**Fig. 4:**
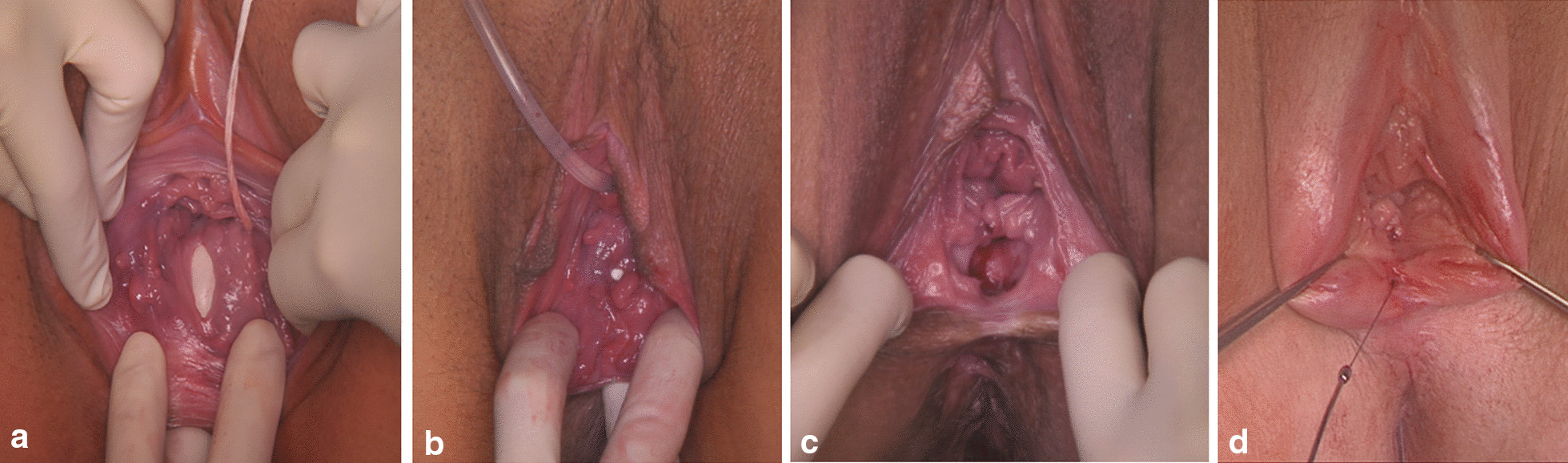
Obstetric rectovaginal fistulas (**a–d**), a and b during a subsequent pregnancy

Twenty-nine out of 40 women suffered from RVF. The patients’ characteristics are summarized in Tables 1 and 3. The size of the RVF varied between 2 and 40 mm in diameter (Fig. 4). Two women presented multiple RVFs (two and three fistulas). Ten RVFs involved the external anal sphincter.

**Table 3:** Characteristics of patients with rectovaginal fistulas

Patient number	Fistula characteristics (diameter, location)	Mode of delivery leading to fistula formation	Age at time of fistula diagnosis (years) and parity	Previous treatment	Fistula treatment in our department
1	2 mm, suprasphincteric	6/2017 spontaneous delivery FGM	28, 2 para		12/2019 vaginal fistula excision, tension-free multilayer closure during a subsequent delivery per cesarean section
2	10 mm, suprasphincteric	12/2017 spontaneous delivery	37, 1 para	12/2017 protective ileostomy	2/2018 vaginal fistula excision, tension-free multilayer closure 8/2018 LIFT (ligation of intersphincteric fistula tract) 12/2018 ileostomy retrocession
3	20 mm, suprasphincteric	2011 spontaneous delivery	24, 1 para	06.01/2017 protective sigmoid colostomy, vaginal and transperineal fistula closure and anal sphincter reconstruction 18.01/2017 vaginal fistula closure 5/2017 transperineal fistula closure 8/2015 transperineal fistula closure	7/2018 vaginal fistula excision, tension-free multilayer closure and Martius-flap interposition 12/2018 colostomy retrocession
4	10 mm, suprasphincteric	4/2016 spontaneous delivery with third-degree perineal tear	25, 1 para		7/2018 vaginal fistula excision, tension-free multilayer closure during a subsequent delivery per cesarean section
5	5 mm, suprasphincteric	2003 spontaneous delivery	27, 1 para		5/2018 vaginal fistula excision, tension-free multilayer closure and Martius-Flap interposition (development of an anoperineal fistula)
6	3 fistulas a 3 mm, suprasphincteric and transsphincteric	12/2016 spontaneous delivery with fourth-degree perineal tear	31, 1 para	12/2016 protective sigmoid colostomy 12/2016 revision of the sigmoid colostomy 2/2017 closure of the sigmoid colostomy and protective transverse colostomy, fistula closure using a transanal rectal-mucosa flap	6/2017 conversion of the rectovaginal fistulas to a fourth-degree perineal tear, fistulectomy, tension-free multilayer closure, anal sphincter reconstruction, levatorplasty, perineoplasty 10/2017 transverse colostomy retrocession
7	15 mm, transsphincteric	9/2011 spontaneous delivery with fourth-degree perineal tear	24, 1 para	two vaginal fistula closure in Libya	5/2013 protective ileostomy, conversion of the rectovaginal fistula to a fourth-degree perineal tear, fistulectomy, tension-free multilayer closure, anal sphincter reconstruction, levatorplasty, perineoplasty 8/2013 ileostomy retrocession
8	5 mm, suprasphincteric	2000 forceps-assisted vaginal delivery	21, 1 para		7/2013 vaginal fistula excision, tension-free multilayer closure and anal sphincter repair
9	20 mm, suprasphincteric	8/2013 spontaneous delivery with fourth-degree perineal tear	37, 2 para	8/2013 vaginal fistula closure	8/2013 protective ileostomy, conversion of the rectovaginal fistula to a fourth-degree perineal tear, fistulectomy, tension-free multilayer closure, anal sphincter reconstruction, levatorplasty, perineoplasty 4/2014 ileostomy retrocession
10	10 mm, suprasphincteric	6/2012 spontaneous delivery with fourth-degree perineal tear	38, 2 para	10/2012 and 2/2013 fistula closure	9/2013 vaginal fistula excision, tension-free multilayer closure
11	3 mm, transsphicteric	7/2012 vacuum-assisted vaginal delivery with fourth-degree perineal tear	30, 1 para	9/2013 transanal rectal-mucosa flap	12/2014 conversion of the rectovaginal fistula to a fourth-degree perineal tear, fistulectomy, tension-free multilayer closure, anal sphincter reconstruction, levatorplasty, perineoplasty during a subsequent vaginal delivery
12	10 mm, transsphincteric	5/2014 spontaneous delivery with fourth-degree perineal tear	28, 1 para	5/2014 vaginal fistula closure	8/2014 conversion of the rectovaginal fistula to a fourth-degree perineal tear, fistulectomy, tension-free multilayer closure, anal sphincter reconstruction, levatorplasty, perineoplasty
13	4 mm, transsphincteric	2006 spontaneous delivery with third-degree perineal tear	30, 3 para		11/2014 conversion of the rectovaginal fistula to a fourth-degree perineal tear, fistulectomy, tension-free multilayer closure, anal sphincter reconstruction, levatorplasty, perineoplasty 3/2015 fistulectomy and tension-free multilayer closure 9/2016 protective ileostomy 9/2016 transanal rectal-mucosa flap (persistent 2 mm suprasphinteric rectovaginal fistula)
14	3 mm, suprasphincteric	1/2013 spontaneous delivery with fourth-degree perineal tear	28, 1 para		8/2015 vaginal fistula excision, tension-free multilayer closure during a subsequent delivery per cesarean section
15	40 mm, suprasphincteric	6/2015 vacuum-assisted vaginal delivery with a button hole tear	31, 1 para		8/2015 vaginal fistula excision, tension-free multilayer closure
16	4 mm, suprasphincteric	5/2018 spontaneous delivery with third-degree perineal tear	30, 1 para	6/2018 protective transverse colostomy 9/2018 and 10/2018 vaginal fistula closure	3/2019 vaginal fistula excision, tension-free multilayer closure 8/2019 transverse colostomy retrocession
17	20 mm, transsphincteric	6/2018 vacuum-assisted vaginal delivery	34, 1 para	19.9/2018 fistulectomy, fistula closure with biomesh interposition and anal sphincter reconstruction 28.09.2018 protective descending colostomy	1/2019 conversion of the rectovaginal fistula to a fourth-degree perineal tear, fistulectomy, tension-free multilayer closure, anal sphincter reconstruction, levatorplasty, perineoplasty 5/2019 descending colostomy retrocession
18	25 mm, suprasphincteric	2/2017 vacuum-assisted vaginal delivery	26, 1 para	2/2017 vaginal fistula closure	2/2019 vaginal fistula excision, tension-free multilayer closure during a subsequent delivery per cesarean section
19	2 fistulas a 3 mm, transsphincteric	3/2017 vacuum-assisted vaginal delivery	30, 1 para	3/2017 and 4/2017 vaginal fistula closure	12/2018 conversion of the rectovaginal fistula to a fourth-degree perineal tear, fistulectomy, tension-free multilayer closure, anal sphincter reconstruction, levatorplasty, perineoplasty during a subsequent cesarean section
20	3 mm, suprasphincteric	2016, spontaneous vaginal delivery	32, 1 para		3/2019 vaginal fistula excision, tension-free multilayer closure during a subsequent delivery per cesarean section
21	10 mm, subsphincteric	11/2016 spontaneous vaginal delivery	32, 1 para	1/2017 fistula closure	4/2019 perineal fistula excision, tension-free multilayer closure and anal sphincter reconstruction during a subsequent delivery per cesarean section
22	2 mm, transsphincteric	1990, spontaneous delivery with third-degree perineal tear	24, 1para		6/2019 LIFT (ligation of intersphincteric fistula tract)
23	15 mm, suprasphincteric	9/2004 spontaneous delivery	30, 1 para	10/2004 and 11/2004 fistula closure	5/2006 protective ileostomy 11/2006 vaginal fistula excision, tension-free multilayer closure 3/2007 ileostomy retrocession
24	20 mm, transsphincteric	8/2011 spontaneous delivery with fourth degree perineal tear	29, 1para		10/2011 vaginal fistula excision, tension-free multilayer closure and anal sphincter reconstruction
25	20 mm, suprasphincteric	10/2008 vacuum-assisted vaginal delivery	32, 1 para		10/2008 protective ileostomy spontaneous fistula closure 2/2009 ileostomy retrocession
26	4 mm, suprasphincteric	2004 spontaneous delivery	25, 1 para		11/2009 vaginal fistula excision, tension-free multilayer closure 5/2010 vaginal fistula excision, tension-free multilayer closure (2 mm persistant rectovaginal fistula)
27	3 mm, suprasphincteric	2000 forceps-assisted vaginal delivery	31, 4 para		3/2008 vaginal fistula excision, tension-free multilayer closure
28	2 mm, transspincteric	9/2008 spontaneous vaginal delivery with third-degree perineal tear	29, 1 para		7/2010 conversion of the rectovaginal fistula to a fourth-degree perineal tear, fistulectomy, tension-free multilayer closure, anal sphincter reconstruction, levatorplasty, perineoplasty during a subsequent vaginal delivery
29	2 mm, suprasphincteric	6/2017 vacuum-assisted vaginal delivery with third-degree perineal tear	26, 1 para		3/2020 spontaneous fistula closure during a subsequent pregnany and delivery per cesarean section

Sixteen out of 29 RVFs were primary fistulas, and 13 were recurrent fistulas that occurred after one to four previous repair attempts at another hospital.

A temporary protective stoma for the diversion of the faecal stream was required in 10 out of the 29 patients; six received an ileostomy, and four received a colostomy.

Twenty-six out of 29 RVFs were closed successfully. After the failed repair one RVF resulted in an anoperineal fistula after fistulectomy and tension-free multilayer closure and Martius flap interposition. The second RVF led to a very small persistent fistula after two fistulectomies and tension-free multilayer closure. Both women are still living with the RVFs. The  third RVF resulted in a smaller RVF after conversion to a fourth-degree perineal tear, a fistulectomy and tension-free multilayer closure and a transanal rectal-mucosa flap and was closed in another hospital. Two out of 29 RVFs closed spontaneously, one (2 mm) during a subsequent pregnancy and one (20 mm) after a protective ileostomy (postpartum) (Table 3).

In seven women, the surgery was carried out via a vaginal approach in the context of a subsequent caesarean section; in two women, surgery was performed following a subsequent vaginal delivery. This procedure was chosen for women at high risk of recurrence or postoperative functional discomfort (vaginal stenosis). All these patients were very slim, had very poor tissue for repair and desired another child. After a spontaneous vaginal delivery, the perineal tear in one case and the small episiotomy in the other case were converted to a fourth-degree perineal tear. The RVFs that were operated on in the context of a caesarean section were repaired by fistulectomy and multilayered closure or a conversion to a fourth-degree perineal tear. All RVFs were successfully closed in the context of a subsequent delivery (Table 3).

## Discussion

The cause of OGF in developing countries is usually a long obstructed labour, and the most common injury is GUF [5]. The authors’ experience shows that in developed countries, OGF occurs after obstetric injuries during both caesarean sections and vaginal deliveries. The most common obstetric fistula in developed countries is the RVF. Browning et al., in their retrospective study in Ethiopia, described the occurrence of VVF in 933 (88.3%) out of 1057 women with obstetric fistulas; 79 (7.5%) out of 1057 had VVF combined with RVF, and 45 (4.3%) had an isolated RVF. Only four (0.4%) women had isolated RVFs that could be confidently attributed to prolonged obstructed labour; the remaining RVFs were due to either sexual or accidental trauma, iatrogenic injury or other causes [5]. Injuries to the pelvis during obstructed labour occur in the low anterior vaginal wall, due to the compression of the foetal head against the pubic symphysis, and the high posterior vaginal wall, due to compression of the foetal head against the sacrum [3]. In our study, the majority of the GUFs had a high anterior location, and all RVFs had a low posterior location. Obstetric urinary trauma can be divided into low or high urinary fistulas. Low fistulas are traditionally caused by ischaemic necrosis as a result of obstructed labour (prolonged compression of the lower vagina, urethra, and bladder base between the foetal head and the symphysis pubis). High juxtacervical, intracervical, or ureteric fistulas usually follow operative interventions, such as caesarean section. Low fistulas can also follow a successful caesarean section performed to relieve obstruction in cases of tissue necrosis in the lower vagina [6].

In our study, we observed one urethro-vaginal fistula after a spontaneous delivery. The 26-year-old patient had given birth to her first child. The urethro-vaginal fistula was likely caused by a tear in the anterior vaginal wall. In seven cases, GUF (VVF with or without involvement of the uterus and vesico-uterine fistulas) was caused by caesarean section alone or combined with a hysterectomy. In a few cases, bladder injury during caesarean section was described. The reason for the utero-vesical fistula that occurred after a spontaneous delivery and persisted for 38 years is unclear. The patient had complained of urinary incontinence since she had given birth to her second child.

One VVF with involvement of the cervix uteri occurred after a forceps-assisted vaginal delivery. In this case, the patient had had a previous caesarean section, and the fistula may have been caused a rupture of the uterine scar with involvement of the cervix-vagina and the bladder. The cause of the vesicovaginal-uterine fistula with detachment of the anterior part of the cervix after spontaneous delivery of the first child remains unclear. It is known that women with previous caesarean sections are at an increased risk of iatrogenic injury [7].

The RVFs in our cohort are attributable to failed perineal tear repair, poor surgical techniques, infection, and wound breakdown. RVF occurs in less than 1% of all vaginal deliveries [8]. According to the literature, a third-degree or fourth-degree perineal tear occurs in 5% of deliveries, of which 1–2% will develop RVF [9]. In Germany, in 2018, the incidences of fourth-degree perineal tears after spontaneous deliveries of singletons and forceps- or vacuum-assisted singleton deliveries were 0.09% (417/466.028) and 0.46% (239/51.611), respectively [10]. Unfortunately, it is not known how many perineal tears result in fistulas in Germany.

In our cohort, GUF was more common in multiparous women (9/11), and RVF was more common in primiparous women (24/29). In two cases, the RVF occurred after a vaginal delivery preceded by a caesarean section, and three RVFs occurred after one to three previous vaginal deliveries.

While the GUFs were all successfully closed (11/11), RVF closure was achieved in 88.65% (26/29). Our results are in line with the published rates of 80–97% for successful surgical closure of obstetric fistula [11–13].

In 10 women with RVF who had a very thin perineum, very poor tissue condition, and a narrow vagina and were consequently at high risk of recurrence or postoperative functional discomfort (e.g., vaginal stenosis, dyspareunia) and who desired another child, we recommend fistula repair in the context of a subsequent delivery. For the first time, pregnancy-related changes in the vaginal wall were used to optimize the success rate of fistula closure. Pregnancy-related changes in the vaginal wall could offer great advantages for fistula closure. In addition to the increased vascularization of the vagina with typical violet coloration during pregnancy (Chadwick sign), the vagina loosens, the vaginal mucosa increases in thickness, and the smooth muscle component of the vaginal wall hypertrophies. The vaginal surface appears velvety [14]. Furthermore, actinonin, a non-specific matrix metalloprotease inhibitor, improves recovery of the parturient vaginal wall after obstetrical injury [15]. One RVF (2 mm) closed spontaneously during a subsequent pregnancy. Seven RVFs were successfully closed simultaneously with the subsequent caesarean section, as were two RVFs following the subsequent vaginal birth.

Symptomatic fistulas produce varying degrees of distress in women. Some RVFs may not need treatment immediately. Therefore, when considering treatment, physicians must weigh the risk and consequences of treatment against the patients’ symptoms.

Although most surgeons agree that continuous urine drainage is important to allow tension-free healing of the surgical scar, opinions vary regarding the length of time that a bladder catheter should be left in situ. In January 2018, the World Health Organization (WHO) released new guidance on the duration of bladder catheterization after the surgical repair of simple obstetric urinary fistulas [16]. The systematic review concluded that a shorter (up to 10 days) duration of bladder catheterization is not associated with significant differences in outcomes when compared with a longer duration of catheterization [17]. A simple fistula is a mid-anterior vaginal wall fistula with minimal scarring and a diameter of 3 cm or less. As the GUFs in our cohort were complex fistulas, we chose a longer catheterization time. The use of a protective stoma is controversial, studies investigating its value are lacking, and there are no guidelines regarding when a stoma should be used [18]. In our opinion, patients are likely to benefit from stool diversion to optimize local healing conditions if significant destruction of the anal canal has occurred, if the RVF is large or if the RVF is recurrent or persistent.

The present observational study specifically examined obstetric-related fistulas in a developed country. The study was limited in that the number of women treated was small, the design was retrospective, and follow-up was early in some cases. The follow-up duration was up to 14 years, depending on when the fistula repair was performed. Nevertheless, to our knowledge, our study is the study with the largest number of patients and describes the management of OGF for both GUF and RVF in a developed country.

## Conclusion

The choice of OGF repair methods should be tailored to the underlying pathology, the type of previous repair, the patients’ wishes and the surgeon’s experience. Fistula repair should be performed after the resolution of the local inflammation, infection and oedema of the tissue surrounding the fistula. When considering treatment, physicians must weigh the risk and consequences of treatment against the patients’ symptoms. Women who suffered from rectovaginal fistulas and were at high risk of recurrence or postoperative functional discomfort and desired another child, the fistula repair should be recommended in the context of a subsequent delivery. If the fistula was tethered so high that its upper edge could not be reached transvaginally, repair should take place via the abdominal route or a combined approach. Flaps and bioimplant interposition should be used for large, recurrent or residual GUFs.

The treatment of genital fistulas in specialized (multidisciplinary) centres is clearly beneficial, as the best chance for fistula closure is at the time of the first operation. Attention should be directed towards preventive obstetric practices and adequate perinatal care, e.g., careful rectovaginal examination after vaginal delivery and the application of adequate surgical techniques when perineal injury occurs. This should be followed by constant care during the postpartum period. Although VVF is rare, in cases of urinary incontinence after pregnancy and delivery, efforts should be made to rule out a VVF.

## Data Availability

The datasets used and/or analysed during the current study are available from the corresponding author on reasonable request.

## Abbreviations

OGF: Obstetric genital fistulas
GUF: Genitourinary fistulas
VVF: Vesico-vaginal fistulas
RVF: Rectovaginal fistulas
